# Significance and Transformation of 3-Alkyl-2-Methoxypyrazines Through Grapes to Wine: Olfactory Properties, Metabolism, Biochemical Regulation, and the HP–MP Cycle

**DOI:** 10.3390/molecules24244598

**Published:** 2019-12-16

**Authors:** Xianfang Zhao, Yanlun Ju, Xiaofeng Wei, Shuo Dong, Xiangyu Sun, Yulin Fang

**Affiliations:** 1 College of Enology, Heyang Viti-Viniculture Station, Northwest A&F University, Yangling 712100, China; zhaoxianfang@nwsuaf.edu.cn (X.Z.); juyanlun2016@nwsuaf.edu.cn (Y.J.); weixiaofeng1993@163.com (X.W.); phoebedong56@gmail.com (S.D.); 2 Life School of Science and Technology, Henan Institute of Science and Technology, Xinxiang 453003, China

**Keywords:** 3-alkyl-2-methoxypyrazines, *Vitis vinifera*, wine, *O*-methyltransferase, *O*-demethylation

## Abstract

3-Alkyl-2-methoxypyrazines (MPs) contribute to the herbaceous flavor characteristics of wine and are generally considered associated with poor-quality wine. To control the MPs in grapes and wine, an accurate understanding of MP metabolism is needed. This review covers factors affecting people in the perception of MPs. Also, the history of *O*-methyltransferases is revisited, and the present review discusses the MP biosynthesis, degradation, and biochemical regulation. We propose the existence of a cycle between MPs and 3-alkyl-2-hydropyrazines (HPs), which proceeds via *O*-(de)methylation steps. This cycle governs the MP contents of wines, which make the cycle the key participant in MP regulation by genes, environmental stimuli, and microbes. In conclusion, a comprehensive metabolic pathway on which the HP–MP cycle is centered is proposed after gaining insight into their metabolism and regulation. Some directions for future studies on MPs are also proposed in this paper.

## 1. Introduction

Volatile 3-alkyl-2-methoxypyrazines (MPs) are a category of off-odor compounds found in wine. MPs contribute to the herbaceous (“vegetal”) characteristics of wine and have extremely low odor detection thresholds, that is, at the level of nanograms or even picograms per liter, as well as very high odor activity [1,2,3]. Excessive herbaceous odor in wine is often thought to be associated with unripe grapes or poor-quality wine [4]. In contrast, low MP levels will decrease the grape varietal characteristic of red wine, and could not balance out the passionfruit and grapefruit odors of volatile thiols in Sauvignon blanc wine from New Zealand [5]. Thus, the MP level is critical to wine quality.

Additionally, the MP level in wine affects customers’ likes and dislikes. A survey was conducted on consumer preference for key aroma compounds. Interestingly, the results showed that consumers with less wine knowledge prefer a wine with a high “green” character, the dominant effect of 3-*iso*butyl-2-methoxypyrazine (IBMP), while people with medium and high wine knowledge pay more attention to the fruity aroma [6]. Also, the origin of MPs affects people’s preference. For example, wines containing 3,5-dimethyl-2-methoxypyrazine (DMMP), one of the MPs often present in wine contaminated by cork stoppers, are generally evaluated negatively [7]. In contrast, wine spiked with 50 ng/L DMMP is noted by the aromas of cherries and red berries [8]. In brief, the presence of MPs in bottled wine is associated with people’s judgement.

**Figure 1:**
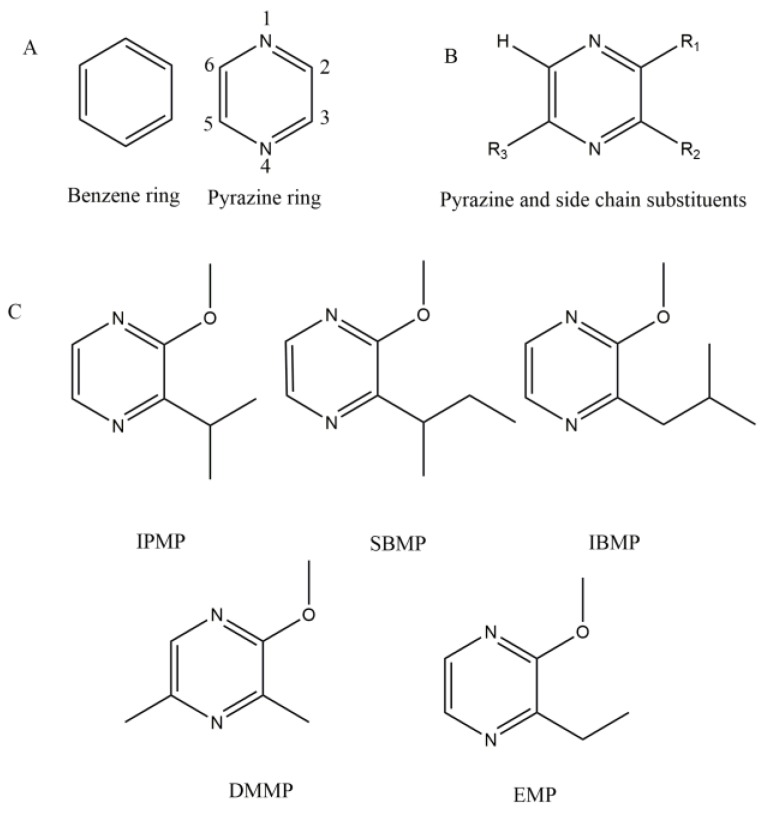
The structure of pyrazines and methoxypyrazines. (**A**) The comparison of the benzene and pyrzine ring. (**B**) The general structure of pyrazines in grapes and wine. (**C**) 3-Alkyl-2-methoxypyrazines found in grapes and wine. IPMP: 3-*iso*propyl-2-methoxypyrazine, IBMP: 3-*iso*butyl-2-methoxypyrazine, SBMP: 3-*sec*butyl-2-methoxypyrazine, EMP: 3-ethyl-2-methoxypyrazine, DMMP: 3,5-dimethyl-2-methoxypyrazine.

Although their effects may appear subjective, the olfactory attributes of MPs are principally determined by objective factors. The chemical structures of pyrazines all share a heterocyclic ring and different side chains (Figure 1). The side chains and their steric and electrostatic effects have been demonstrated to be responsible for their unique aromatic properties [9,10,11]. When MPs are released from wine or other foods and enter the nasal cavity, they specifically interact with odorant-binding proteins (OBPs) [12,13,14]. Then, specific information from the molecular structures of the MPs is delivered to the brain [14,15,16]. Clearly, this physiological process is quite objective, but subjective factors are involved in the processing and perception of that information by the brain. The outcome of the brain processing is conditioned by personal history, environmental variables, subjective covariates, and the characteristics of the object [15]. It is possible that the human genetic differences in the receptors are an important factor as well, but no reports touch on this field. 

A very broad spectrum of the flavor impressions for MPs was summarized after our extensive data collecting. These flavor impressions range from veggie to woody to some off-odors. To avoid confusion and chaos resulting from the wide-ranging flavor impressions of MPs, the first goal of this paper was to comprehensively review the affecting factors and then propose some suggestions.

The MPs in wine mainly originate from grapes. These compounds are found in numerous grape varieties, such as Sauvignon blanc, Chardonnay, Semillon, Riesling, Cabernet Sauvignon, Merlot, Cabernet franc, and Carménère [17,18,19]. MPs have been shown to be present in all tissues of the grape such as skins, seeds, and flesh [20,21]. In addition, those MPs can be regulated by many practical managements (i.e., defoliation, light exposure, irrigation, temperature, and so on) [21,22,23,24,25,26,27] to satisfy customers. Considering the high odor activity of MPs and their relatively low levels in grapes and wine, accurate regulation of MPs is required. Thus, the second goal of this review was to summarize the development of MP metabolism and biochemical regulation and to suggest regulatory aims for future studies.

The metabolic pathways for MPs have been discussed for almost 50 years, but all of the steps, except for *O*-methylation, remain ambiguous [28]. *Vitis vinifera* *O*-methyltransferases (VvOMTs) catalyze the *O*-methylation step, which consists of the transformation of 3-alkyl-2-hydroxypyrazines (HPs) into the corresponding MPs [29,30,31,32]. However, only a few reports have focused on the degradation mechanisms of MPs in *V. vinifera*. In this paper, we attempt to propose a metabolic cycle between HPs and MPs. The cycle is important for controlling the MP levels in grapes and wine. 

The conversion between HPs and MPs is the basis of their regulation. For example, defoliation can decrease MP contents, while not affecting *VvOMT* expression [33]. Is the MP decreasing caused by the photodegradation or the activation of an undefined enzyme at the *O*-demethylation step? After discussion, we think that the *O*-demethylation enzyme is more reasonable than the photodegradation. Therefore, the establishment of clear and complete metabolic and regulatory pathways for MPs is necessary and will be beneficial to future studies and the wine industry.

## 2. Molecular Structure and Sensory Properties

### 2.1. Structure

Pyrazine is a heterocyclic aromatic organic compound with the chemical formula of C_4_N_2_H_4_. Compared with a benzene ring, the pyrazine has two nitrogen atoms replacing the carbon atoms at positions 1 and 4 (Figure 1A). The size of the pyrazine ring is similar to that of the benzene ring. Moreover, like the carbon atoms of the benzene ring, the four carbon atoms and two nitrogen atoms of the pyrazine ring constitute a rigid plane. The carbon atoms at positions 2, 3, 5, and 6 in the pyrazine ring are active in chemistry and can be substituted by other chemical groups in chemical synthesis processes. Regarding the volatile MPs in grape berries and wine, only a hydrogen atom substituent has thus far been found occupying position 6 of the pyrazine ring (Figure 1B). Generally, a methoxy group (-O-CH_3_) is linked to the C-2 position of the pyrazine ring (R1 in Figure 1B). 

Except for DMMP, the difference between other MPs lies in the substituents at the C-3 site, as indicated by their names (Figure 1C). At the C-3 site, IBMP has an isobutyl group; 3-*iso*propyl-2-methoxypyrazine (IPMP), an *iso*propyl group; 3-*sec*butyl-2-methoxypyrazine (SBMP), and a *sec*butyl group; and 3-ethyl-2-methoxypyrazine (EMP), an ethyl group. DMMP has two methyl- groups: one is located at the C-3 site, and the other is located at the C-5 site. 

### 2.2. Diverse Odor Descriptions

**Figure 2:**
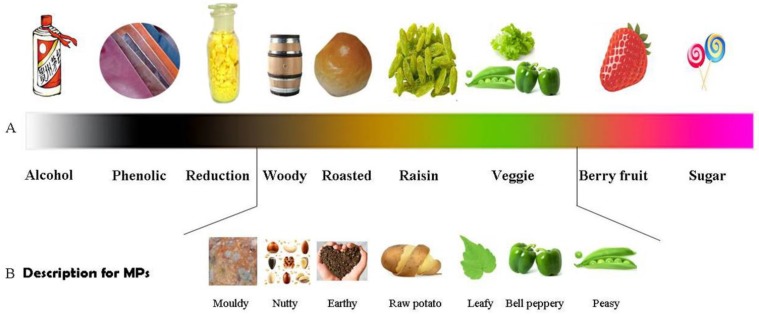
The spectrogram of MPs’ impressions. (**A**) Drawn according to the classification of Escudero et al. [34]. (**B**) The collected descriptions for MPs.

The odors of wine were classified as alcohol, phenolic, reduction, woody, roasted, raisin, veggie, berry fruit, and sugar according to Escudero et al. [34] (Figure 2). The spectrum of the flavor impressions for MPs ranges from veggie (bell pepper, peas, ivy leaves, potato) [3,4,14], to woody (earthy, cocoa-like) [4,18,35], to chemical (caramel, aldehydic) [3], to some off-odors (moldy, wet cardboard) [3,36]. MPs cover over half of the whole spectrum. Therefore, the diverse descriptions of the characteristic of MPs may cause confusion in communication. In fact, the odorant impression has a certain objectivity based on the following three factors.

The first is the varied steric and electrostatic properties of the compounds. When the number of carbon atoms in the R2 substituent (Figure 1B) is greater than two, the pyrazine derivative shows a strong tendency to have a bell-pepper aroma, and when the group is a hydrogen atom, a methyl group, and ethyl group, that is, has fewer than two carbon atoms, the volatile substance smells nutty [10]. Yoshii and Hirono [11] compared 54 conformers of nine pyrazine derivatives and found that the steric and electrostatic features were responsible for the green odor, which agrees to the results of Wagner et al. [9]. Therefore, the chemical structure is the determiner of the olfactory attributes of MPs. 

The second factor is the concentration of MPs. IBMP at a low concentration (2–8 ng/L) is described as musty. As the IBMP concentration increases to 8–16 ng/L, it smells like green pepper. When IBMP is at a concentration of 16–64 ng/L in a model wine, it smells leafy [2,37]. Clearly, the concentration of MPs closely affects people’s sensory verdict of a wine.

**Figure 3:**
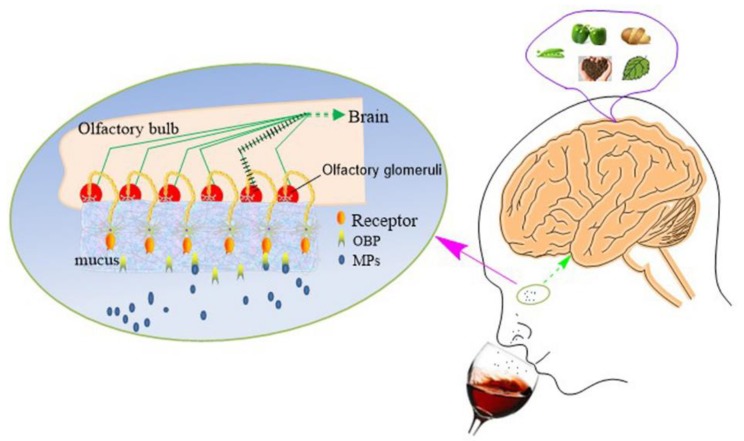
The schematic diagram on the process of the perception and description of MPs. MPs (blue dots and cycles) are sniffed into the nasal cavity. They are combined with the olfactory binding proteins (OBP, light yellow ellipses with blue tails). The complex of MPs–OBP is across the mucus and binds with the receptors (orange ellipses with tails). Then, the signal is transduced to the brain along the activated signal pathway (green lines with black bars). The impressions are generated after brain processing.

The verdict is perhaps also related to the third factor: the physiology of the olfactory system (Figure 3). When air as well as the aroma compounds enter the nose and reach the olfactory mucosa, the OBPs, a subclass of lipocalins, can bind IBMP [14]. The results of IBMP molecular dynamics simulations show that the ligand is buried into the OBP cavity [12] and that the binding stabilizes the tertiary structure of porcine OBP-I [13]. Those OBP–IBMP complexes cross a layer of mucus, bind with the olfactory receptors [14,38], and activate a signal transduction pathway [16]. Ultimately, the visual information forms in the brain. However, the outcome of brain processing is conditioned by personal history, environmental variables, subjective covariates, and the characteristics of an object [15]. For example, DMMP isolated from the cork stopper-contaminated wine is evaluated negatively [7]; in contrast, wine spiked with DMMP with 50 ng/L DMMP is noted by the aromas of cherries and red berries [8]. The judgments on DMMP are diametrically opposed to each other, indicating that some subjective factors function while people are judging. 

Overall, although the odor impressions for MPs are determined by some objective factors, such as the molecular structures and the concentration of MPs, as well as partially by the physiology of the olfactory system, the flavor description is also impacted by subjective factors from the test panel. In tasting tests, a small panel of 8–12 people is generally used [39,40]. This panel size, though those panelists are well trained, appears to be too small. The International Standards on the sensory anlaysis in the quality control, to our best knowledge, have not defined the size of the tasting panel. Yet, there is a file (ISO 11136) on the hedonic tests with consumers in a controlled area demanding that the size of the tasting panel never be less than 60 [41].

### 2.3. Extremely Low Odor Detection Threshold

The detection of MPs requires special analytical methods owing to the presence in wine at nanogram per liter levels [42]. Amazingly, MPs at nanogram per liter levels in wine can be perceived by nose. The extremely low odor detection thresholds (Table 1) with quite high odor potency highlight the importance of MPs in the evaluation of wine quality [3]. 

Analogous to the diverse odor descriptions of MPs, their thresholds are affected by the varied steric and electrostatic properties of pyrazines and the olfactory physiology. In addition to the two factors mentioned, the matrix in which MPs are dissolved is another important element. After comparing the data listed in Table 1, we found that the effect of the matrix generally ranked from low to high as follows: air < water < white wine < synthetic wine < red wine. Wine, as is well known, regularly contains ethanol, sugars, acids, tannins, anthocyanins, and volatile compounds, among other components. As they are all hydrophobic organic chemicals, MPs tend to bind with those wine ingredients, especially ethanol. Ethanol promotes the dissolution of MPs into the aqueous phase [43,44], which makes the threshold of MPs in wine higher than that in air and water. Another impacting factor is drawn our attention. The same compounds could also be different as they were determined either via orthonasal or retronasal under the perception of DMMP (Table 1) [8].

In addition, owing to the combination of subjective factors, the precise determination of the odor detection threshold appears to be difficult [51]. To solve this problem, some mathematical models have been introduced. In brief, an array of pyrazines is employed to evaluate the thresholds. The raw data obtained are used to establish mathematical models with bilinear regression [16], multiple linear regression [52], and artificial neural networks [10]. All of the three established models show a high correlation between the pyrazine structures and odor detection thresholds. Provided that the side chains are given, the precise threshold can be predicted by the models. Obviously, these models do not consider the steric and electrostatic properties of the molecules [9,11]. Because of this deficiency, there is still much work left to be done on the precise prediction of the odor detection threshold.

## 3. Metabolic Pathways for MPs in Grapes

As a class of volatile compounds, MPs outweigh the other aroma compounds in wine evaluation owing to their exceptional olfactory attributes. To guarantee wine quality, the content of MPs in wine should be rigidly controlled. As most MPs originate from grapes, the biosynthetic pathway of MPs has been an area of focus. In contrast, the reverse pathway, biodegradation, has almost completely remained unclear. Omitting the study of biodegradation and putting emphasis only on MP biosynthesis is misleading and detrimental to the wine industry. 

### 3.1. Biosynthesis

#### 3.1.1. Precursors to and Formation of the Pyrazine Ring

**Figure 4:**
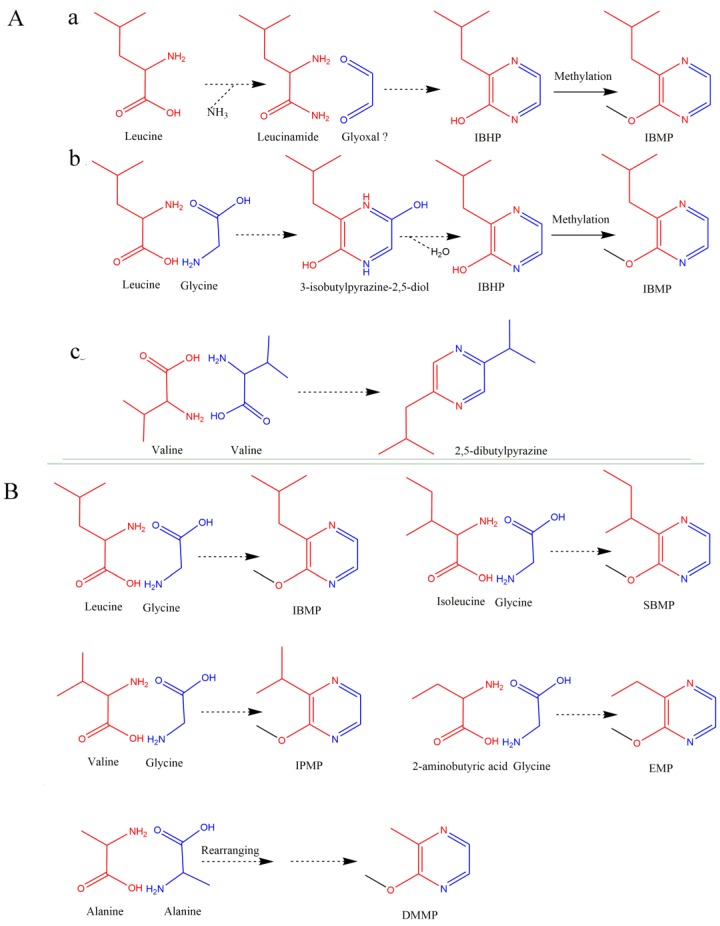
The proposed biosynthesis pathways of pyrazines (**A**) and the hypothesized pathway and precursors for 3-alkyl-2-methoxypyrazines in grapes and wine (**B**). (**A-a**) is from Murray et al. [46], (**A-b**) from Cheng et al. [55], and (**A-c**) from Nawrath et al. [56]. The colors of red and blue display the origination of hemical groups. “Glyoxal?” means that the chemical *glyoxal* is questioned. IBHP: 3-*iso*butyl-2-hydroxypyrazine.

**Figure 5:**
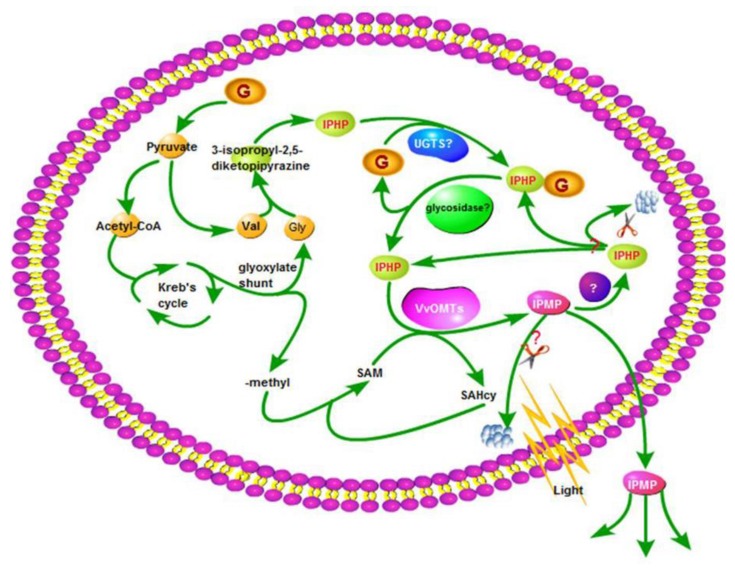
The schematic diagram on the proposed biosynthesis and biodegradation of 3-*iso*propyl-2-methoxypyrazine (IPMP). G: glucose; Val: valine; Gly: glycine; IPHP: 3-*iso*propyl-2-hydroxypyrazine; UGTS: UDP-glucuronosyltransferase; SAM: *S*-adenosyl-l-methionine; SAHcy: *S*-adenosylhomocysteine; VvOMT: *Vitis vinifera* *O*-methyltransferases.

The pyrazine ring is a six-membered heterocycle whose first and fourth sites are occupied by nitrogen atoms (Figure 1A). The studies on the formation of such heterocycle are often seen in microbials. In 1970, Murray et al. [46] proposed an IPMP biosynthetic pathway starting from leucine and glyoxal (Figure 4(Aa)). This proposal was questioned by Nursten and Sheen [53] for the following reasons: (i) there is no evidence showing that amino acids can form *α*-amino acid amides, and (ii) the presence of glyoxal is hardly found. Another pathway was proposed and validated with the isotope labeling approach [54]. Cheng et al. [55] confirmed these results and proposed a novel and widely-accepted biosynthetic pathway for IPMP (Figure 4(Ab) and Figure 5) that is consistent with the results of Nawrath [56] (Figure 4(Ac)). 

Studies on the formation of the pyrazine ring in plants are scarcely found. On an Académie Amorim in 2003, Roujou de Boubée [57] issued the studies on IBMP in grapes and wines. He demonstrated that the addition of leucine in the media could promote the IBMP biosynthesis by the undifferentiated callus of Cabernet Sauvignon. The finding is line with the results of a latest study conducted by Lei and colleagues [58]. They observed that L-leucine treatment on Cabernet Sauvignon grapes could significantly increase the IBMP content and the related gene expression of *VvOMT1* and *VvOMT3*. Therefore, the two teams speculated that L-leucine may be one of the precursors of IBMP. However, the addition of stable isotopes (L-leucine-d_10_ and ^15^NH_4_Cl) did not lead to isotopic enrichment of the IBMP produced by the Cabernet Sauvignon callus [57]. Lei et al. [28] explained that the stable isotope was difficult to track accurately. It appears to be not a convincing explanation, because both the research teams believe in the proposal of Murray et al. [46]. In this proposal, NH_4_Cl and leucine should produce leucinamide, and the leucinamide as well as glyoxal would be condensated into the pyrazine ring. However, it seems that the stable isotope labelled NH_4_Cl was not involved into the reaction, and that the D atom from L-leucine-d_10_ might be substituted during the condensation (Figure 4A,B). 

In all, it is more promising to explain the MP biosynthesis with the proposal of Cheng et al. [55] than with that of Murray et al. [46]. Therefore, it is an assumption that the precursors of IPMP, IBMP, SBMP, EMP, and DMMP are valine and glycine, leucine and glycine, isoleucine and glycine, 2-aminobutyric acid and glycine, and two alanines, respectively (Figure 4B).

#### 3.1.2. States of HPs in Grapes

After the formation of the pyrazine ring, an enolization follows, resulting in the generation of HPs [54]. Ryona et al. [59] observed a strong inverse correlation between 3-*iso*butyl-2-hydroxypyrazine (IBHP) and IBMP for Cabernet Franc. Nevertheless, Harris et al. [60], the same research team, denied the observation that IBHP concentrations increase proportionally to the decrease in IBMP in wine grapes, when improving the detection method for IBHP. In fact, the trend of 3-*iso*butyl-2-hydroxypyrazine (IBHP) is similar with that of IBMP as grape developing, but with some days’ delay [60]. This trend is confirmed by another study conducted by Dunlevy et al. [30]. In Cabernet Sauvignon grapes, the highest concentration occurs around the sixth week post flowering, and the level of IBHP is about 40-fold higher than that of IBMP. In all, IBHP exists in grape berries, even in those varieties (i.e., Pinot Noir) that are not capable of producing IBMP [30]. 

So, which is the state of the IBHP in the pool, free, or in combination with some ligand? Both conjugated and unconjugated IBHPs are present in the urine of rats at 24 h after feeding with IBMP [61]. This result encouraged Ryona and colleagues [59], who found a significant increase (33%) in IBHP in Cabernet franc after acid hydrolysis, which indicates that IBHP exists partially in a bound state in grapes. However, Harris et al. [60] re-evaluated that hypothesis by assessing acid hydrolysis in CF1 grapes and observed a slight, yet nonsignificant (*p >* 0.05) elevation in IBHP levels. The conjugation is possibly related to the grape varieties. To verify this hypothesis, the testing of more varieties is required. 

Another question is the identity of the ligand. Among the candidates, glucose has the most potential. On the one hand, glycosylated IBHP has been identified in animals [61]. On the other hand, aromatic alcohols (e.g., 4-methylguaiacol, guaiacol, and cresols) tend to combine with glycosyl groups [62]. Because of these two reasons, Ryona et al. [59] thought that a portion of IBHP is in the form of IBHP–glycosyl. However, they chose acids instead of β-glucosidase to hydrolyze the samples [59]. An increase in the IBHP concentration after β-glucosidase-mediated hydrolysis would strongly support the combination of IBHP with glucose (Figure 5). 

#### 3.1.3. O-Methylation

Under the catalysis of *O*-methyltransferase (OMT), HPs accept the “-CH_3_” and transform into MPs. A *VvOMT* gene was identified by Hashizume et al. [63]. The purified protein, VvOMT, held the function of *O*-methylation. However, the enzyme showed stronger specific activity for caffeic acid than for IBHP or IPHP. In 2010, another two *VvOMT* genes were firstly identified [29], and were named *VvOMT1* and *VvOMT2* owing to their function in the *O*-methylation step. The catalytic efficiency of VvOMT1 was found to be higher than that of VvOMT2 because of the differences in equivalent residues in the active sites of VvOMT1 (F319, L322) and VvOMT2 (L319, V322) [29,64]. Another two genes were identified three years later [30,31] and were termed *VvOMT3* and *VvOMT4*. The VvOMT3 enzyme displays a higher affinity for IBHP than VvOMT1 and VvOMT4 [30]. Thus, *VvOMT3* is proposed as the major gene involved in IBMP biosynthesis in grapevines [30,65]. In latest years, *OMT* genes in *Coffea arabica* [66] and *Nicotiana benthamiana* [32] were also found by blast against *VvOMTs*. 

The functions of *VvOMT* genes are all verified by gene recombination in *Escherichia coli*. The purified proteins hold the capability of *O*-methylation of HPs in vitro. Surprisingly, the combinants of *VvOMT* genes also hold the function of *O*-methylation in vivo, because IBMP was detected in the fermentation broth used to culture the VvOMT combinants [31]. However, the homologous verification of the *O*-methylation has not been conducted to date. 

The relation between gene expression and MP biosynthesis to some extent accounts for *VvOMT* gene function. The pattern of *VvOMT* gene expression over time generally mirrors MP biogeneration, that is, increasing before veraison and then decreasing [30,33,67]. However, there is a one- to three-week delay between the *VvOMT* expression peak and the highest IBMP concentration [30,33]. At all points, *VvOMT* gene expression is consistent with the MP levels in grapes.

Although VvOMT has been verified to possess the function of *O*-methylation, VvOMT has been demonstrated to be a multifunctional enzyme [63]. Interestingly, the Vmax values for IBHP and caffeic acid under the catalysis of VvOMT are 0.73 pkat/mg and 175 pkat/mg, respectively, suggesting that the optimal catalytic substrate of VvOMT is caffeic acid, not IBHP [63]. Also, the proteins VvOMT1, VvOMT2, and VvOMT4 show greater activity against other compound such as quercetin, orcinol, isoeugenol, and so on [29,30]. VvOMT3 protein exhibits the highest specific activity against IBHP and IPHP, but the affinity against eugenol and isoeugenol is quite high (isoeugenol: 641 ± 45 pkat/mg, eugenol: 415 ± 25 pkat/mg) [29]. To explain this observation, we have to seek help from other OMT models in plants.

**Figure 6:**
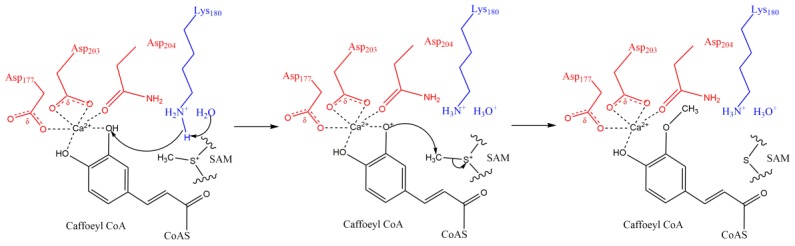
Proposed catalytic reaction mechanism of SbCCoAOMT (redrawn according to Walker et al. [73]).

*O*-methylation is common in plants. The proton at the hydroxyl group of various substrates can be substituted by a methyl group (Figure 6). The structures of the substrates and the catalytic mechanism of OMTs in plants share the following similar properties:

(i) The SAM binding regions, which often comprise the amino acid residues (Tyr, His, Asp, Thr, Ser, and so on), are highly conserved [68,69];
(ii) At the active site, divalent cations (Mg^2+^ [69,70,71] or Ca^2+^ [72,73]) are necessary;
(iii) The substrate molecule has one or more cyclic group (benzene or pyrazine ring, Figure 1A). On the ring, there are often at least two branched pendant groups: a hydroxyl group, which serves as the methyl receptor; and an assistant group that binds to Mg^2+^ or Ca^2+^, the catalytically active center, by ionic bonding [74].

Once the three aforementioned factors are satisfied, the *O*-methylation reaction will take place in plant cells. To illustrate the mechanism of OMT catalysis, we employed the model of *Sorghum bicolor* caffeoyl–CoA *O*-methyltransferase (SbCCoAOMT, Figure 6), the best-studied *O*-methylation plant model. During the reaction catalyzed by SbCCoAOMT [73], a calcium ion located at the active site captures caffeoyl–CoA and SbCCoAOMT by ionic bond formation. While SAM is associated with SbCCoAOMT, the binding results in a significant conformational change, which leads to the Lys-180 residue on the enzyme approaching the hydroxyl methyl acceptor. With the coordination of a water molecule, Lys-180 subtracts a proton from the hydroxyl group. Then, the S-methyl group of SAM is nucleophilically attacked by the oxyanion, thereby resulting in the transfer of the methyl group from SAM. At this point, SAM and the product feruloyl–CoA are released, and the *O*-methylation step is completed. In this model, the divalent cation Ca^2+^ occupies the active sites and nonspecifically binds to the substrate and certain amino acid residues in the enzymes, which may be the reason that OMTs have been shown to be capable of methylating more than one substrate [63,74].

### 3.2. Release and Biodegradation

The MP levels in clusters tend to decrease dramatically in maturity [75]. There are three possible fates of MPs in vivo: (i) storage in grape berries, (ii) secretion into the air, and (iii) biodegradation (Figure 5). 

In intact grape berries, MPs seem to be retained and then change with phenological phases. For example, IBMP levels in grape fruits generally accumulate before veraison, reach a maximum at veraison, and then decrease markedly with ripening until harvest [30,60,75]. The residual-free MPs in harvested grapes will be transferred into the must and wine. 

MPs are released into the air when grape berries are damaged. There is circumstantial evidence that lady beetles prefer to feed on previously damaged berries versus undamaged fruits [76], because MPs are aggregation pheromones for these insects [77,78]. 

The biodegradation pathway of MPs is hypothesized to involve enzymatic demethylation back into HPs in grapes. There is a balance between HPs and MPs in grapes; that is, HPs are both the precursor and the degradation products of MPs [60]. *O*-demethylation may be the first step of the biodegradation pathway for MPs [59]. There is some collateral evidence for this hypothesis. Hawksworth and Scheline [61] observed that IBHP was detected in urine and urinary metabolites 24 h after rats were fed with IBMP. In humans and animals, alkyl groups on the pyrazine ring are transformed into hydroxyl groups under the catalysis of P450-type enzymes or into carboxyl groups by molybdenum-containing oxidases of the xanthine oxidase type [61,79,80,81]. In *V. vinifera*, VvDIOX-like (Genebank Accession: CAO70478), an uncharacterized protein, might possess the function of *O*-demethylation according to blast and cluster analysis [82]. 

### 3.3. HP–MP Cycle

**Figure 7:**
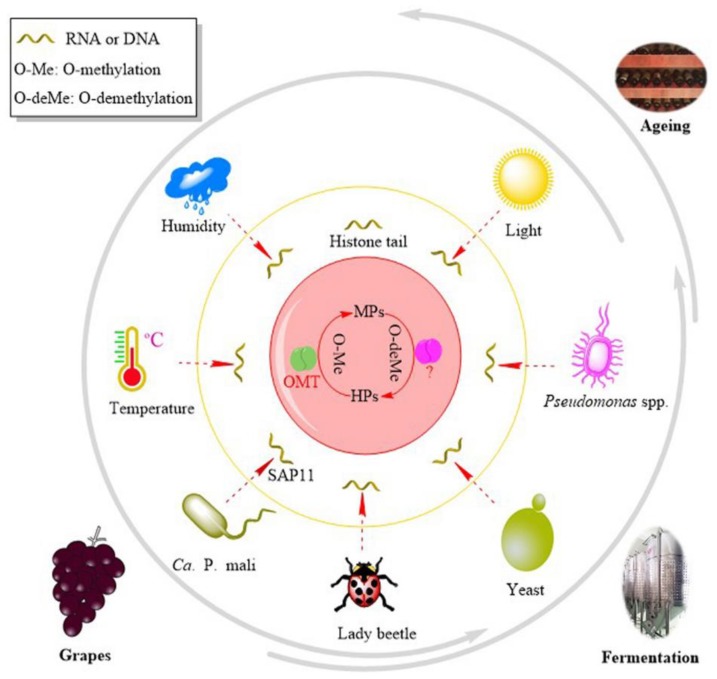
The schematic diagram on the gene regulations and the factors impacting MPs’ levels from grapes to wine aging.

The above discussion indicates that HPs can be transformed to MPs via *O*-methylation and that the reverse reaction, *O*-demethylation, also exists. The two processes constitute a small HP–MP cycle (Figure 5 and Figure 7). As MPs are associated with wine quality, the primary role of the cycle is the control of MP levels. Regarding this cycle, putting emphasis only on MPs’ generation is misleading. Equally, MP degradation is important as well. The quantities of HPs that have not been converted into MPs should also been considered. Assuming that they are carried into must and wine, those HPs can be methylated into corresponding MPs by yeasts during alcohol fermentation [83]. In short, the HP–MP cycle is theoretically the starting point to control MP contents in grapes and wine.

## 4. Regulation

The conversion between MPs and the corresponding HPs via *O*-methylation and *O*-demethylation (Figure 5 and Figure 7) appears to be regulated by some abiotic and biotic factors such as genes, environmental stimuli, light, temperature, humidity, fertilizers, lady beetles, and microbials. 

### 4.1. Gene Regulation

Gene regulation covers a wide range of mechanisms that are employed by cells to change the production of specific gene products (protein or RNA). It includes DNA modification and regulation of transcription, post-transcription, and translation. Owing to the limited information on gene regulation of *VvOMT* locus expression in grapevines, only transcriptional regulation is described herein. 

Recently, a *Jittery*-like transposable element was found to be located on the upstream sequence of the *VvOMT3* locus [65]. The *Jittery*-like transposable element has the potential to explain the loss of *VvOMT3* expression by an epigenetic silencing mechanism [84]. Battilana et al. [65] focused on the silencing regulation of *VvOMT3* by histone tail modification. The results show that the cross-talk between the different types of the histone tail modification is important for the quality trait of berries because of the spatial and developmental regulation of *VvOMT3* expression. Phytoplasma SAP11 can also suppress the biosynthesis of IBMP in *Nicotiana benthamiana* [32]. SAP11 is one kind of virulence effector secreted by the Aster yellows witches’-broom phytoplasma strain. The expression of *SAP11* blocks *NbOMT1* expression in *N. benthamiana* [32]. These two examples show that changes in the gene *OMT* have a direct impact on the production of MPs. On the one hand, this result confirms the function of the enzyme OMT and, on the other hand, it implies the role of the HP–MP cycle. When OMT is blocked, the pathway from HPs to MPs is interrupted. Subsequently, MP levels decrease owing to the mechanisms of *O*-demethylation.

### 4.2. Environmental Stimuli

In viticultural practices, the dynamics and regulation of MP biosynthesis and accumulation in grapevines are key to controlling MP levels in wine [25]. *VvOMT* gene expression and the dynamic changes in MPs in response to several management measures, such as light exposure [30]; leaf removal and light exposure [33]; and sunlight restriction, lateral shoot removal, and water deficit [67], have been investigated. However, the relationship between environmental factors and the gene regulatory sequences has not been established. To control MP levels in grapes and wine, it is necessary to reinforce the studies on interaction between the gene *VvOMT3* and its upstream regulatory elements.

#### 4.2.1. Light 

IBMP is a light-sensitive compound [85]. It is strongly correlated with the light intensity in the canopy [86]. Grapes exposed to full light have one-third of the IBMP concentration of berries covered by three leaf layers [87]. The exposure of clusters to light before veraison can reduce the accumulation of IBMP [23,59], or may promote the photodegradation of IBMP [30], but is unable to increase post-veraison degradation [59]. Thus, light may be involved in the gene regulation of *O*-demethylation before veraison. As red grape berries mature, the anthocyanins accumulate in skins; those pigments have a high absorption luminous radiation in the visible region [88]. It is possible that light via anthocyanins is involved in the IBMP degradation. This supposition is supported by the results of *VvOMT* expression. Defoliation is an effective measure to increase light exposure and to decrease MP content in grapes. However, the *VvOMT1*, *VvOMT2*, and *VvOMT3* gene expression in Sauvignon blanc grapes has been shown to be unaffected [33]. Considering that sunlight decreases the IBMP levels in grapes [89], it is reasonable that sunlight has a function in the *O*-demethylation pathway of the HP–MP cycle.

However, it is important to note that the sun emits light accompanied by temperature. An attempt to explore the influence of light was conducted by Plank [90,91], who utilized a cold light illuminator (light-emitting diodes, LEDs) to separate the effects of light exposure from those of heat. The result showed that light exposure rather than temperature is most significant in preventing greater MP accumulation in grape berries. 

The concentrations of MPs during wine aging are also affected by light. At one year, the MP concentrations in wine in a clear bottle and a green- or amber-colored bottle exposed to light decline approximately 60% and 40% faster, respectively, than those in wine stored in darkness, which shows potential for reducing MP levels. Nevertheless, fluorescent and UV lights do not affect the MP concentrations in wine [92,93].

#### 4.2.2. Temperature

Compared with the effect of light, the effect of temperature on MPs in grapes appears to be minor. In addition, there has not been an experiment designed specifically to explore the impact of temperature. On the one hand, the influence of temperature is estimated by subtracting the light effect from the sunlight effect, as mentioned above. On the other hand, the temperature effect is also indicated by comparing the MP concentrations in grapes and wines from cool regions with those in grapes and wines from warmer regions. Generally, IBMP levels in wines from cooler regions are higher than those in wines from warmer regions [25,75,86,87,94]. In addition, the MPs from the cluster stems are shown to be eliminated over 95% by stream treatment (100 °C, 60 min) of red wine must solution [95]. It is possible that high temperature promotes the emission and degradation of MPs from must and wine.

#### 4.2.3. Rainfall, Humidity, and Irrigation

To investigate the effect of rainfall on MP levels, Mendez-Costabel and colleagues [96] utilized a plastic tarpaulin to cover the ground during the entire dormant season. A weekly crop evapotranspiration rate of 70% was maintained until commercial harvest by irrigation. The biosynthesis of IBMP is significantly greater in vines under normal rainfall conditions than in covered vines [96]. Additionally, rainfall leads to high humidity. In humid vintages, the grapes generate a higher IBMP content throughout the ripening period, even though the sunshine is stronger and the temperature is higher [4].

Similarly, too much irrigation, especially during the later mature period, will result in wines with the herbaceous odors of green peppers [97,98]. This defective odor is related to higher IBMP levels originating from grapes and entering wines [24]. Wines made from grapes treated with minimal irrigation are rated significantly lower in terms of vegetal and bell-pepper aroma than those from grapes irrigated under standard conditions [99]. Stress induced by a combination of lateral shoot removal and water deficit (T-3) was conducted to detect the changes in *VvOMT*. The results show that the timing of *VvOMT3* mRNA expression in developing seeded berries is associated with the period of IBMP accumulation in samples from the T-3 group [67]. In summary, irrigation, especially during grape ripening, will increase the MP contents and lead to striking vegetal or bell-pepper odor.

#### 4.2.4. Nitrogen Fertilizers

As previously illustrated, pyrazines are considered derivatives of cyclic dipeptide anhydrides. Several amino acids are potential biosynthetic precursors. However, Helwi et al. [100] demonstrated that nitrogen status does not directly affect the IBMP levels in grapes, even though recent studies proved that foliar nitrogen applications disturbed the contents of amino acids in grapes, must, and wines [101]. However, Helwi et al. [100] also pointed out that nitrogen fertilizers can promote vine vigor, which subsequently affects the microclimate and MP concentration. 

### 4.3. Biotic Factors

#### 4.3.1. Lady Beetles 

Lady beetles are commonly seen in vineyards all over the word. The adult insects prefer to aggregate on berry clusters, especially skin-damaged grape berries, from 2–3 weeks before the mature period to the harvest [76]. When the beetles are disturbed during the grape harvest or processing, lady beetles release a yellow hemolymph with an unpleasant odor [102]. Additionally, the insect bodies, whether alive or dead, can release very unpleasant aroma compounds into wines [103]. Once released into wine, the causal compounds in the hemolymph fluid will mask the varietal aroma and give rise to undesirable vegetal, herbaceous, bell-pepper, and earthy odors [102,103,104,105]. A total of 38 kinds of volatile chemicals have been isolated and identified [105]. Among those chemicals, IPMP, as well as SBMP, IBMP [102,103,104,105], and DMMP [104,105], is one of the key compounds responsible for the ladybeetle taint (LBT) in wine [102,103,104,105]. 

The odor detection threshold of LBT varies with grape varieties. For example, 10 beetles per liter of Red Bergamais must modified the wine aroma and flavor characteristics significantly [106,107], and 1.9 beetles per kilogram of grapes or 0.27 beetles per kilogram of grapes of Frontenac is estimated as the threshold at which 10% of the population could detect the characteristic off-flavor of *Harmonia axyridis* [108]. For Pinot noir and Riesling, additional approximately 3 beetles per kilogram of grapes and 4 beetles per kilogram of grapes, respectively, are the odor detection thresholds [109]. The tolerance limit for LBT is proposed to be 200–400 beetles per metric ton of grapes [110]. Obviously, beetles pose a high potential threat to the wine industry.

#### 4.3.2. Yeasts and Microbes

Yeast is irreplaceable in wine fermentation. Most commercial yeasts have no capacity of producing MPs [83]. However, Lalvin BM45 yeasts are proved to increase IPMP by 29% in wine fermentation [83]. However, it is hard to determine whether IPMP is directly synthesized by the yeasts or by other microbes under the regulation of the yeasts, as some bacteria can produce IPMP as well. Bokulich et al. [111] found that Pseudomonales are 14.2% of the bacterial profile in California wine must. Some *Pseudomonas* spp., that is, *P. taetrolens* and *P. perolens*, have been proven to be able to generate IPMP [54,55]. Therefore, those microorganisms might have an effect on MP levels in grapes and wine.

## 5. Conclusions and Outlook

Owing to their intense vegetal odors, which are thought to be closely related to unripe grapes and poor-quality wines, MPs are crucial to grapes and wines. The perception of MPs in wine during winemaking and commercial activities is mainly dependent on the olfactory sense. However, the description of MP odors ranges from veggie to woody to some off-odors. This broad spectrum impairs communication on this topic. Thus, expansion of the taste panel and introduction of suitable statistical approaches are recommended. 

The extremely low odor detection threshold and level of MPs in wine indicate the need to rigidly control MP generation in grapes. We established a schematic to account for the biosynthesis and degradation of MPs (Figure 7). MPs are speculated to originate from the condensation of two amino acid molecules, which leads to pyrazine ring formation. Then, HPs are *O*-methylated into the corresponding MPs under the catalysis of VvOMT, and MPs are reversibly transformed into HPs via *O*-demethylation. There exists a metabolic cycle (Figure 5 and Figure 7) between HPs and MPs consisting of *O*-methylation and *O*-demethylation under the catalysis of VvOMT and the enzyme responsible for *O*-demethylation. To control the MP content accurately, the cycle should be strongly considered.

MP metabolism is regulated by genes, environmental stimuli, and microbes. These factors function by the upstream regulatory elements located on the promoter region sequence upstream of the *VvOMT* gene sequences. In this case, more breakthroughs linking practical management and the regulatory elements are expected. Furthermore, studies on the biodegradation of MPs should be strengthened. The key is the identification of the genes responsible for degradation. Fortunately, VvDIOX-like (NCBI Accession: CAO70478), an uncharacterized protein, was identified as a candidate by blast and cluster analysis [82]. Once the related genes are ascertained, regulation will follow. In addition, a complete understanding of the metabolic and regulatory pathways will help offer theoretical guidance for the accurate control of MPs in the future. 

## Figures and Tables

**Table 1:** The olfactory thresholds of 3-alkyl-2-methoxypyrazines in grapes and wine.

Acronym	Threshold (ng/L)	Matrix	Reference
IPMP	2	White wine	[1]
	0.0005–0.001 *	Air	[35]
	1–2	Water	[18,45,46]
	2	Red wine	[2]
	2	Synthetic wine	[2]
IBMP	0.002–0.004 *	Air	[35]
	0.5–16	Water	[2,18,45]
	1	White wine	[1]
	10–16	Red wine	[2,4,18]
	2–6	Synthetic wine	[2,4]
SBMP	1–2	Water	[45]
EMP	425	Water	[47,48]
DMMP	56	Air	[49,50]
	31 **	Red wine	[8]
	70 ***	Red wine	[8]

Note: *: the extreme low concentration in air compared with those in other matrixes may be caused by the amounts of substances: for air, 1 L ≈ 0.045 mol; for water, l L ≈ 55.56 mol. **: the estimate of the orthonasal group; ***: the estimate of the retronasal group. IPMP: 3-*iso*propyl-2-methoxypyrazine, IBMP: 3-*iso*butyl-2-methoxypyrazine, SBMP: 3-*sec*butyl-2-methoxypyrazine, EMP: 3-ethyl-2-methoxypyrazine, DMMP: 3,5-dimethyl-2-methoxypyrazine.

## Abbreviations

DMMP	3,5-dimethyl-2-methoxypyrazine
EMP	3-ethyl-2-methoxypyrazine
HPs	3-alkyl-2-hydroxypyrazines
IBHP	3-*iso*butyl-2-hydroxypyrazine
IBMP	3-*iso*butyl-2-methoxypyrazine
IPHP	3-*iso*propyl-2-hydroxypyrazine
IPMP	3-*iso*propyl-2-methoxypyrazine
LBT	the ladybird taint
MPs	3-alkyl-2-methoxypyrazines
OBP	odorant binding proteins
OMT	*O*-methyltransferase
SAM	*S*-adenosyl-l-methionine
SBMP	3-*sec*butyl-2-methoxypyrazine
UDP	Uridine Diphosphate
